# Trk-fused gene (TFG) regulates pancreatic β cell mass and insulin secretory activity

**DOI:** 10.1038/s41598-017-13432-x

**Published:** 2017-10-12

**Authors:** Takeshi Yamamotoya, Yusuke Nakatsu, Akifumi Kushiyama, Yasuka Matsunaga, Koji Ueda, Yuki Inoue, Masa-Ki Inoue, Hideyuki Sakoda, Midori Fujishiro, Hiraku Ono, Hiroshi Kiyonari, Hisamitsu Ishihara, Tomoichiro Asano

**Affiliations:** 1 Department of Medical Science, Graduate School of Medicine, University of Hiroshima, 1-2-3 Kasumi, Minami-ku, Hiroshima City, Hiroshima, 734-8551 Japan; 2 Division of Diabetes and Metabolism, The Institute for Adult Diseases, Asahi Life Foundation, Chuo-ku, Tokyo, 103-0002 Japan; 3 Center for Translational Research in Infection & Inflammation, School of Medicine, Tulane University, 6823, St. Charles Avenue, New Orleans, LA 70118 USA; 4 Division of Neurology, Respirology, Endocrinology and Metabolism, Department of Internal Medicine, Faculty of Medicine, University of Miyazaki, 5200 Kihara, Kiyotake, Miyazaki, 889-1692 Japan; 5 Division of Diabetes and Metabolic Diseases, Nihon University School of Medicine, Itabashi, Tokyo, 173-8610 Japan; 6 Department of Clinical Cell Biology, Graduate School of Medicine, Chiba University, 1-8-1 Inohana, Chuo-ku, Chiba City, Chiba, 260-8670 Japan; 7 Animal Resource Development Unit and Genetic Engineering Team, RIKEN Center for Life Science Technologies, 2-2-3 Minatojima Minami-machi, Chuou-ku, Kobe, 650-0047 Japan

## Abstract

The Trk-fused gene (TFG) is reportedly involved in the process of COPII-mediated vesicle transport and missense mutations in TFG cause several neurodegenerative diseases including hereditary motor and sensory neuropathy with proximal dominant involvement (HMSN-P). The high coincidence ratio between HMSN-P and diabetes mellitus suggests TFG to have an important role(s) in glucose homeostasis. To examine this possibility, β-cell specific TFG knockout mice (βTFG KO) were generated. Interestingly, βTFG KO displayed marked glucose intolerance with reduced insulin secretion. Immunohistochemical analysis revealed smaller β-cell masses in βTFG KO than in controls, likely attributable to diminished β-cell proliferation. Consistently, β-cell expansion in response to a high-fat, high-sucrose (HFHS) diet was significantly impaired in βTFG KO. Furthermore, glucose-induced insulin secretion was also markedly impaired in islets isolated from βTFG KO. Electron microscopic observation revealed endoplasmic reticulum (ER) dilatation, suggestive of ER stress, and smaller insulin crystal diameters in β-cells of βTFG KO. Microarray gene expression analysis indicated downregulation of NF-E2 related factor 2 (Nrf2) and its downstream genes in TFG depleted islets. Collectively, TFG in pancreatic β-cells plays a vital role in maintaining both the mass and function of β-cells, and its dysfunction increases the tendency to develop glucose intolerance.

## Introduction

The Trk-fused gene (TFG) was initially identified as an oncogene causing thyroid cancer, in which the N-terminal half of TFG was fused with neurotrophic tyrosine kinase receptor 1 (NTRK1, also known as TrkA)^1^. Subsequently, TFG was also reported to be a fusion partner of the anaplastic lymphoma kinase (ALK) gene in anaplastic large cell lymphoma^2^, though the function of TFG itself remained essentially unknown until quite recently.

In 2011, TFG was reported to be localized at endoplasmic reticulum (ER) exit sites (ERES) and to be crucial in transport from the ER to the Golgi apparatus via COPII vesicles^3^. TFG is important for retention of COPII vesicles between the ER and ER-Golgi intermediate compartments (ERGIC), and in the absence of TFG, COPII-coated carriers become dispersed throughout the cytoplasm^4^, although the effect of TFG depletion on protein secretion remains elusive^3–5^.

On the other hand, TFG was recently identified as a causative gene for several neurodegenerative diseases, such as hereditary motor and sensory neuropathy with proximal dominant involvement (HMSN-P)^6–8^, the axonal type of Charcot-Marie-Tooth disease^9^ and hereditary spastic paraplegia (HSP)^10–12^. Among HMSN-P patients, high incidences of diabetes mellitus and dyslipidemia have been reported^13^, yet nothing is known about the role of TFG in the regulation of glucose or lipid metabolism.

To determine whether TFG is involved in insulin secretion, we generated pancreatic β-cell specific TFG knockout (βTFG KO) mice by crossing TFG floxed mice (TFG^loxP/loxP^) with β-cell specific Cre transgenic mice which express Cre recombinase driven by the mouse insulin promotor (MIP-Cre). The βTFG KO mice showed impaired glucose tolerance and insulin secretion upon glucose stimulation *in vivo*, which was attributed to impairments of both the mass and function of β-cells. This is the first study, to our knowledge, using conditional TFG knockout mice and also the first to provide a mechanistic link between TFG and glucose metabolism.

## Results

### β-cell specific TFG knockout mice showed impaired glucose tolerance and insulin secretion *in vivo*

**Figure 1:**
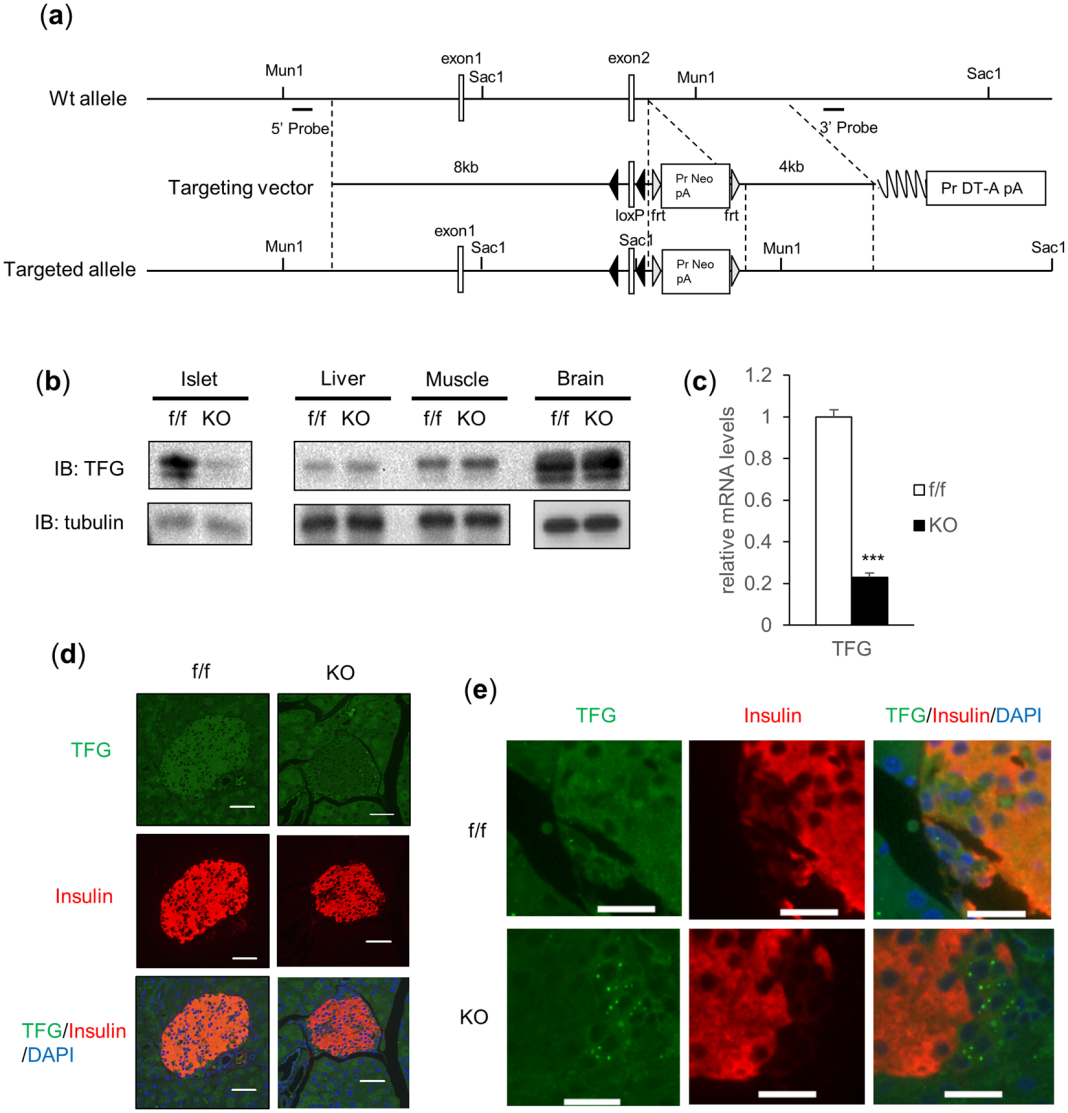
TFG was knocked out specifically in pancreatic β-cells in TFG^loxP/loxP^; MIP-Cre mice. (**a**) The scheme for the genomic construction of TFG floxed mice. (**b**) Immunoblotting of TFG and tubulin in multiple tissues of TFG^loxP/loxP^ (f/f) and TFG^loxP/loxP^; MIP-Cre (KO) mice (8 weeks old). Full-length blots are presented in Supplementary Figure S4. (**c**) mRNA levels of TFG in isolated islets (10-week-old mice, n = 4). (**d**) Pancreatic sections from 9-week-old mice were immunostained with antibodies against TFG (green) and insulin (red). scale bars: 50 μm. (**e**) Enlarged images of TFG and insulin immunostaining in the pancreatic samples from f/f and KO mice. scale bars: 20 μm. ***P < 0.001.

β-cell specific TFG knockout mice (βTFG KO) were generated by crossing TFG^loxP/loxP^ mice (f/f) (Fig. 1a) with MIP-Cre mice. We confirmed that the protein and mRNA levels of TFG were markedly downregulated in the isolated islets of βTFG KO mice (Fig. 1b,c), whereas TFG expression levels were unchanged in other tissues (Fig. 1b). Immunostaining of pancreatic sections with antibodies against TFG and insulin revealed specific depletion of TFG in insulin-positive cells (Fig. 1d,e).

**Figure 2:**
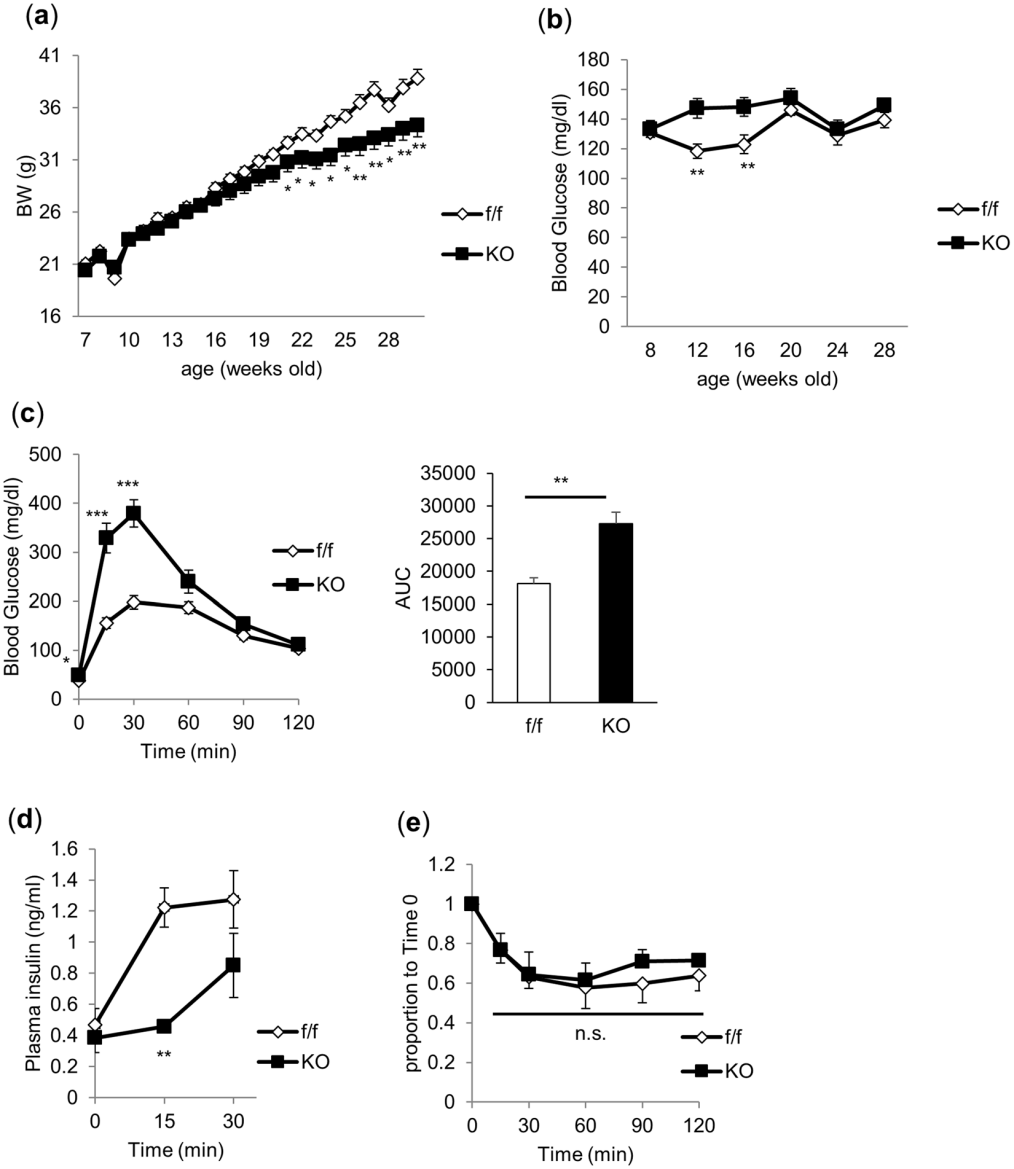
TFG^loxP/loxP^; MIP-Cre mice showed glucose intolerance with impaired insulin secretion. (**a**) Body weight (normal chow, n = 11–12). (**b**) Blood glucose levels (normal chow, n = 11–12). (**c**) Blood glucose levels and area under the curve on IPGTT (9-week-old mice, 2 g/kg i.p., n = 6–7). (**d**) Plasma insulin levels in response to intraperitoneal glucose injection (17 to 19-week-old mice, 2 g/kg i.p., n = 4–5). (**e**) ITT (10-week-old mice, 0.75 U/kg i.p., n = 4–5). *P < 0.05, **P < 0.01, ***P < 0.001.

βTFG KO mice grew, maintaining normal appearances, but their body weights were slightly lower than those of the controls after approximately 20 weeks of age (Fig. 2a). Non-fasting blood glucose levels of βTFG KO mice were slightly but significantly higher at the ages of 12 and 16 weeks, but then returned to the same levels as in the control mice (Fig. 2b). Subsequently, 9-week-old βTFG KO and control mice were subjected to intraperitoneal glucose tolerance tests (2 g/kg) (Fig. 2c). Whereas the glucose levels before glucose administration differed only slightly between f/f and KO mice, βTFG KO mice had markedly higher blood glucose levels after glucose administration, especially at the 15 and 30 min time points, than the controls. Plasma insulin levels 15 min after the glucose injection were significantly lower in KO mice (Fig. 2d). In contrast, the insulin tolerance test (ITT) revealed no significant difference between 10-week-old βTFG KO and control mice (Fig. 2e), indicating impaired insulin secretion to be the cause of the glucose intolerance observed in the βTFG KO mice.

### TFG knockout in β-cells resulted in reduced β-cell mass due to diminished β-cell proliferation

**Figure 3:**
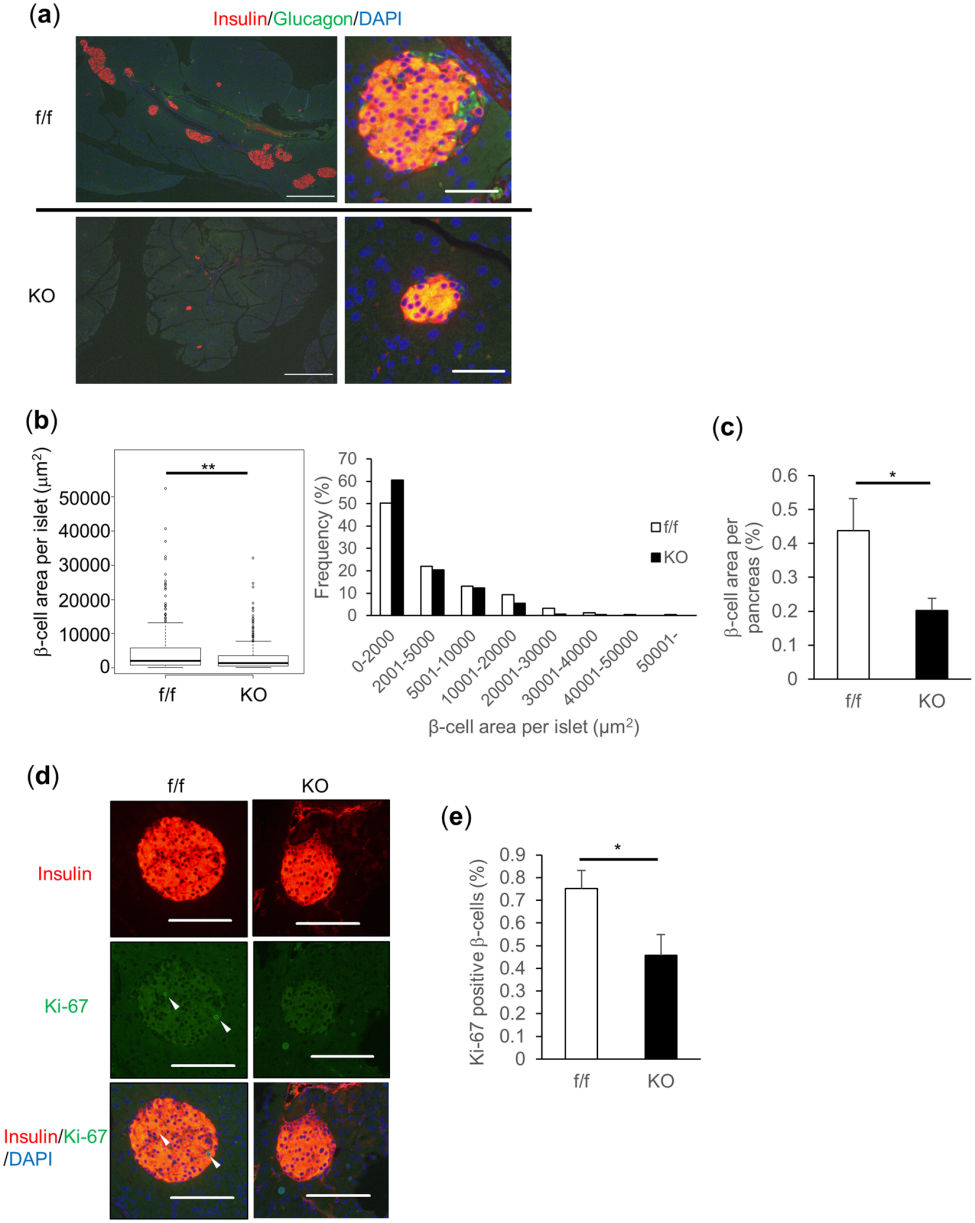
TFG^loxP/loxP^; MIP-Cre mice showed reduced β-cell mass as a consequence of diminished β-cell proliferation. (**a**) Pancreatic sections from 9-week-old mice were immunostained with antibodies against insulin (red) and glucagon (green). scale bars: 500 μm (left panels), 50 μm (right panels). (**b**) Box plot and histogram for β-cell area per islet (f/f: 249 islets from 8 mice, KO: 269 islets from 10 mice, Mann-Whitney U test, **P < 0.01). (**c**) Percentage of β-cell area over total pancreatic area. (**d**) Immunostaining for Ki-67 (green) and insulin (red). White arrowheads indicate Ki-67 positive β-cells. scale bars: 100 μm. (**e**) Percentage of Ki-67 positive β-cells (f/f: 41 positive cells among 6022 β-cells, KO: 24 positive cells among 5006 β-cells). 9-week-old mice, n = 8–10 (**b**–**e**), *P < 0.05, **P < 0.01.

To elucidate the mechanisms of impaired glucose tolerance *in vivo*, β-cell volume was assessed by immunostaining pancreatic sections from βTFG KO and control mice with antibodies against insulin (Fig. 3a). β-cell area per islet and the proportion of β-cell area to the total pancreas were revealed to be significantly smaller in βTFG KO than in control mice (Fig. 3b and c, respectively). Considering that Ki-67 positive β-cell proliferation was significantly lower in βTFG KO than in control mice (Fig. 3d,e), while TUNEL staining detected almost no apoptotic β-cells in either group (data not shown), it is very likely that the reduced β-cell mass observed in βTFG KO mice is attributable to reduced β-cell proliferation. The diminished β-cell proliferation in KO as compared with wild-type mice was also observed at the age of 3 weeks (Supplementary Fig. S1a,b). This difference was more marked than that observed in the 9 week-old mice (Fig. 3e).

**Figure 4:**
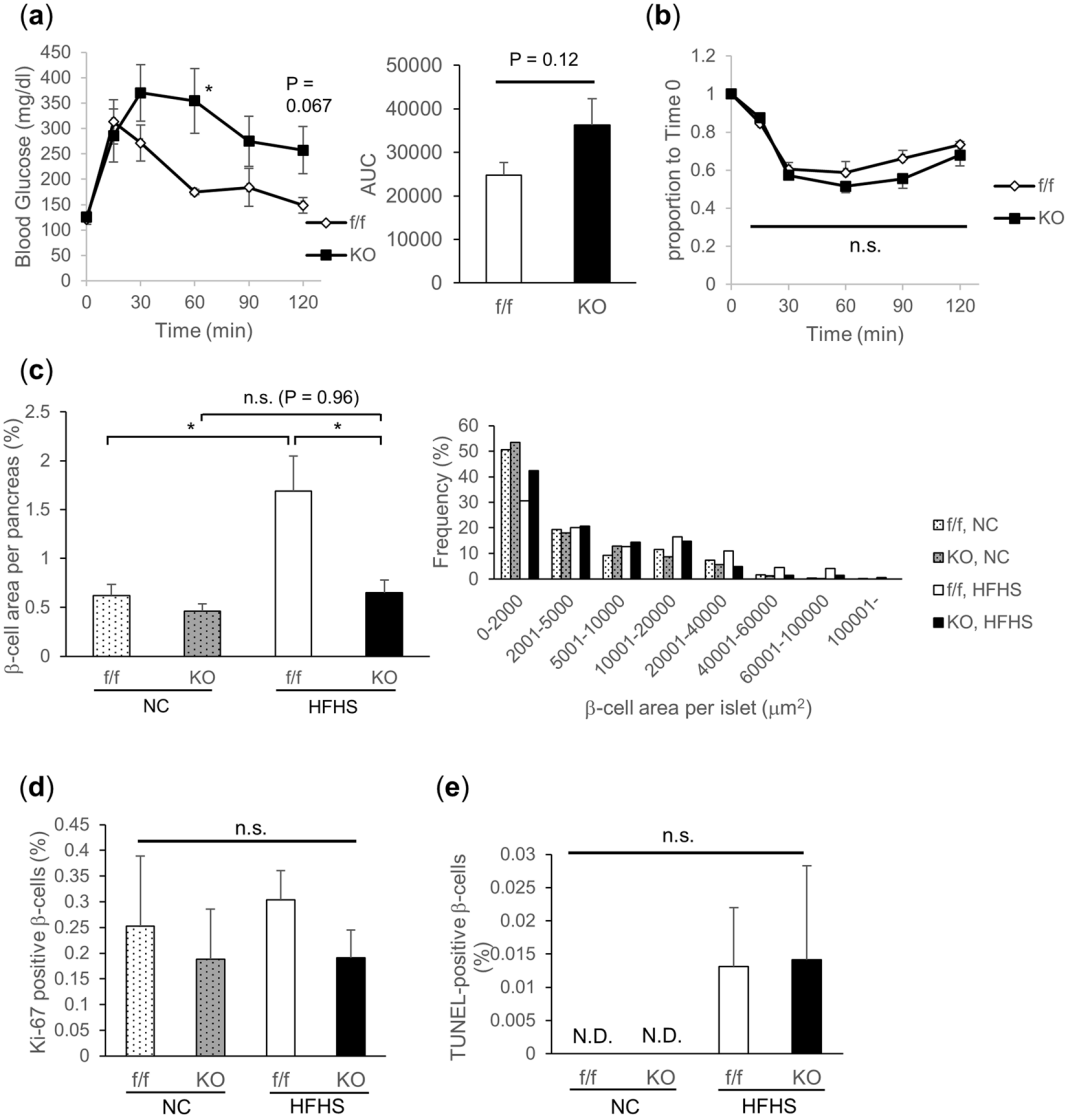
Islet hypertrophy in response to high-fat high-sucrose (HFHS) diet was attenuated in TFG^loxP/loxP^; MIP-Cre mice. (**a**) IPGTT (19-week-old HFHS-fed mice, 2 g/kg i.p., n = 5–6). (**b**) ITT (20-week-old HFHS-fed mice, 0.75 U/kg i.p., n = 5–6). (**c**) Percentage of β-cell area over total pancreatic area and histogram for β-cell area per islet. (**d**) Percentage of Ki-67 positive β-cells (f/f, NC: 16 positive cells among 4760 β-cells, KO, NC: 7 positive cells among 3710 β-cells, f/f, HFHS: 46 positive cells among 15005 β-cells, KO, HFHS: 15 positive cells among 7824 β-cells). (**e**) Percentage of TUNEL positive β-cells. 26-week-old mice, n = 5 for normal chow (NC), n = 6–8 for HFHS (**c**–**e**), *P < 0.05. N.D. not detected.

To further verify the physiological importance of TFG in the regulation of β-cell mass, mice were fed a high-fat, high-sucrose (HFHS) diet, starting at age 6 weeks, for 20 weeks to induce islet hypertrophy which occurs to compensate for insulin resistance. After 13 to 14 weeks of HFHS diet feeding, glucose intolerance was suggested based on a tendency toward increased AUC in the intraperitoneal glucose tolerance test (IPGTT) in βTFG KO mice (Fig. 4a). In contrast, no significant difference was observed in ITT between βTFG KO and control mice (Fig. 4b). After 20 weeks of HFHS diet feeding, while significant islet hypertrophy was observed in the control mice as a compensatory change in response to exacerbation of insulin resistance, βTFG KO mice failed to increase β-cell mass in response to the HFHS diet (Fig. 4c). Of note, the difference in β-cell mass between the genotypes in the normal chow (NC) group was somewhat less marked than that in 9-week-old mice (Fig. 3c). Though not statistically significant, the proliferation of Ki-67 positive β-cells was relatively low in KO mice regardless of whether they were fed the NC or the HFHS diet (Fig. 4d), with no concurrent increase in TUNEL-positive cells (Fig. 4e). These results suggest inadequate proliferation to be the cause of the impaired β-cell mass expansion in response to the HFHS diet, in βTFG KO mice.

### Loss of TFG in β-cells impairs glucose-induced insulin secretion in isolated islets

**Figure 5:**
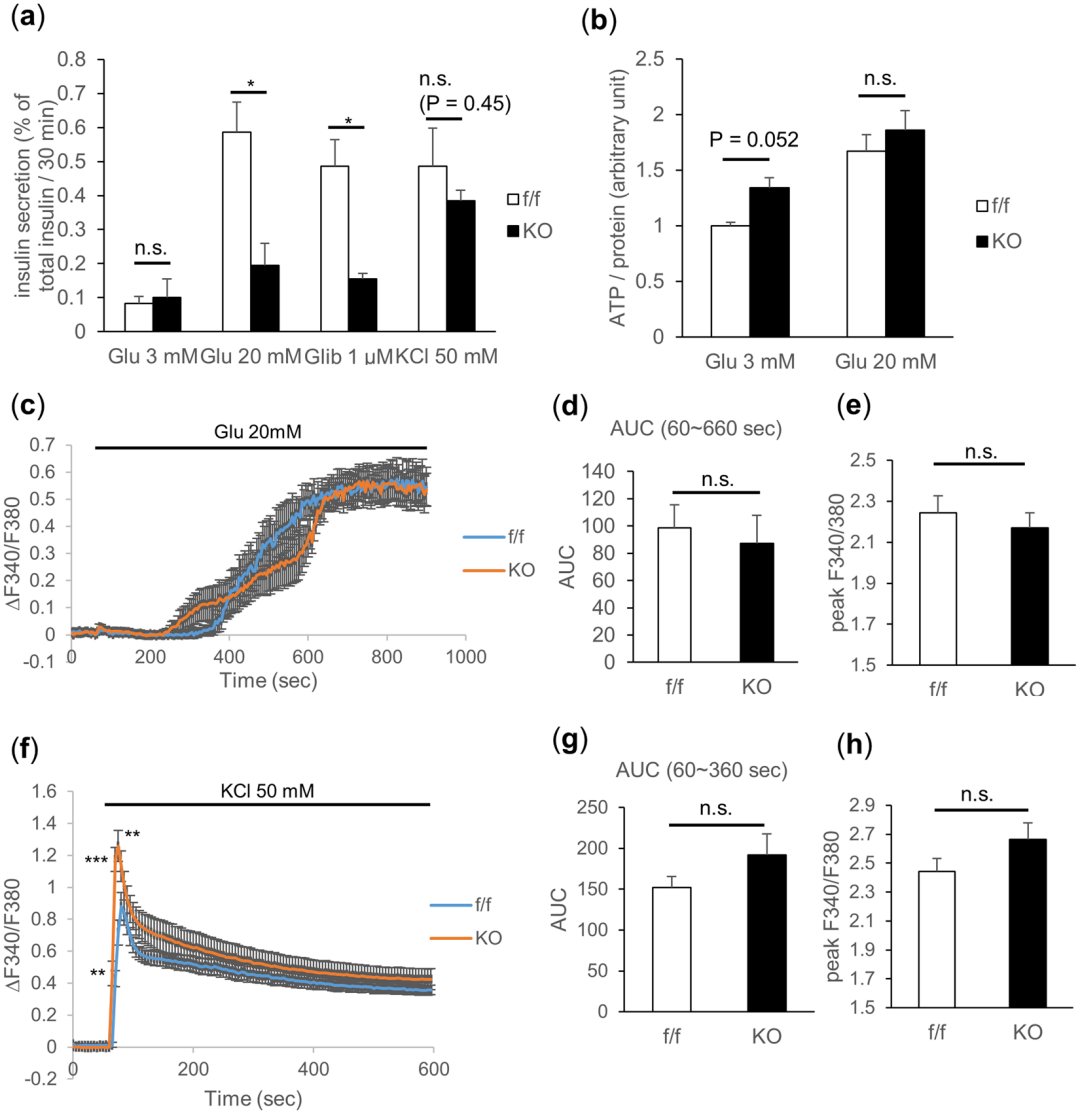
TFG knockout in β-cells severely impaired glucose-stimulated insulin secretion. (**a**) Insulin secretion from isolated islets when stimulated with glucose (3 mM, 20 mM), glibenclamide (1 μM) or KCl (50 mM) for 30 min. The percentage of secreted insulin over islet insulin content (determined from the insulin concentration of the lysates) is shown (10-week-old mice, n = 3–4). (**b**) Islet ATP content (normalized by the protein content, 13-week-old mice, n = 3–5). (**c**–**h**) Analysis of intracellular Ca^2+^ concentration when stimulated with 20 mM glucose (**c**–**e**) or 50 mM KCl (**f**–**h**). (**c**,**f**) The changes in the Fura-2 ratio (ΔF340/F380) from the time of stimulation (60 sec) are demonstrated. The average of 5–7 independent measurements is shown (8 to 10-week-old mice). (**d–g**) Area under the curve during the 10 min (for glucose stimulation) or the 5 min (for KCl stimulation) from the time of stimulation (60 sec). (**e**,**h**) Peak Fura-2 ratio (F340/F380). *P < 0.05, **P < 0.01, ***P < 0.001.

To investigate the effects of TFG depletion on β-cell function, islets isolated from either βTFG KO or control mice were stimulated with insulin secretagogues such as glucose, glibenclamide (sulfonylurea) and KCl. Insulin secretion from βTFG KO islets was severely impaired upon stimulation with high doses of glucose or glibenclamide, whereas the difference was less marked with KCl stimulation (Fig. 5a). Islet ATP concentrations measured before and after glucose stimulation were not lower, instead being slightly higher although not to a statistically significant extent, in βTFG KO islets, which ruled out the possibility of impaired ATP production as the cause of defective insulin secretion in βTFG KO islets (Fig. 5b). Intracellular Ca^2+^ level alterations when islets were stimulated with either a high concentration of glucose (Fig. 5c–e) or KCl (Fig. 5f–h) were also analyzed by fluorescence-based assay using Fura-2 AM. Changes in the Fura-2 ratio (ΔF340/F380) (Fig. 5c), area under the curve (AUC) (Fig. 5d) and the peak Fura-2 ratio (Fig. 5e) showed no significant differences in response to glucose stimulation. The rise in the intracellular Ca^2+^ concentration was not impaired with KCl stimulation (Fig. 5f–h), though ΔF340/F380 was higher at several time points immediately after the stimulation (Fig. 5f). Collectively, these results suggest that the mechanisms underlying the impaired insulin secretion in TFG depleted β-cells are downstream from the rise in the intracellular Ca^2+^ concentration.

### TFG-depleted β-cells have smaller insulin crystals within insulin granules and show mild signs of ER stress

**Figure 6:**
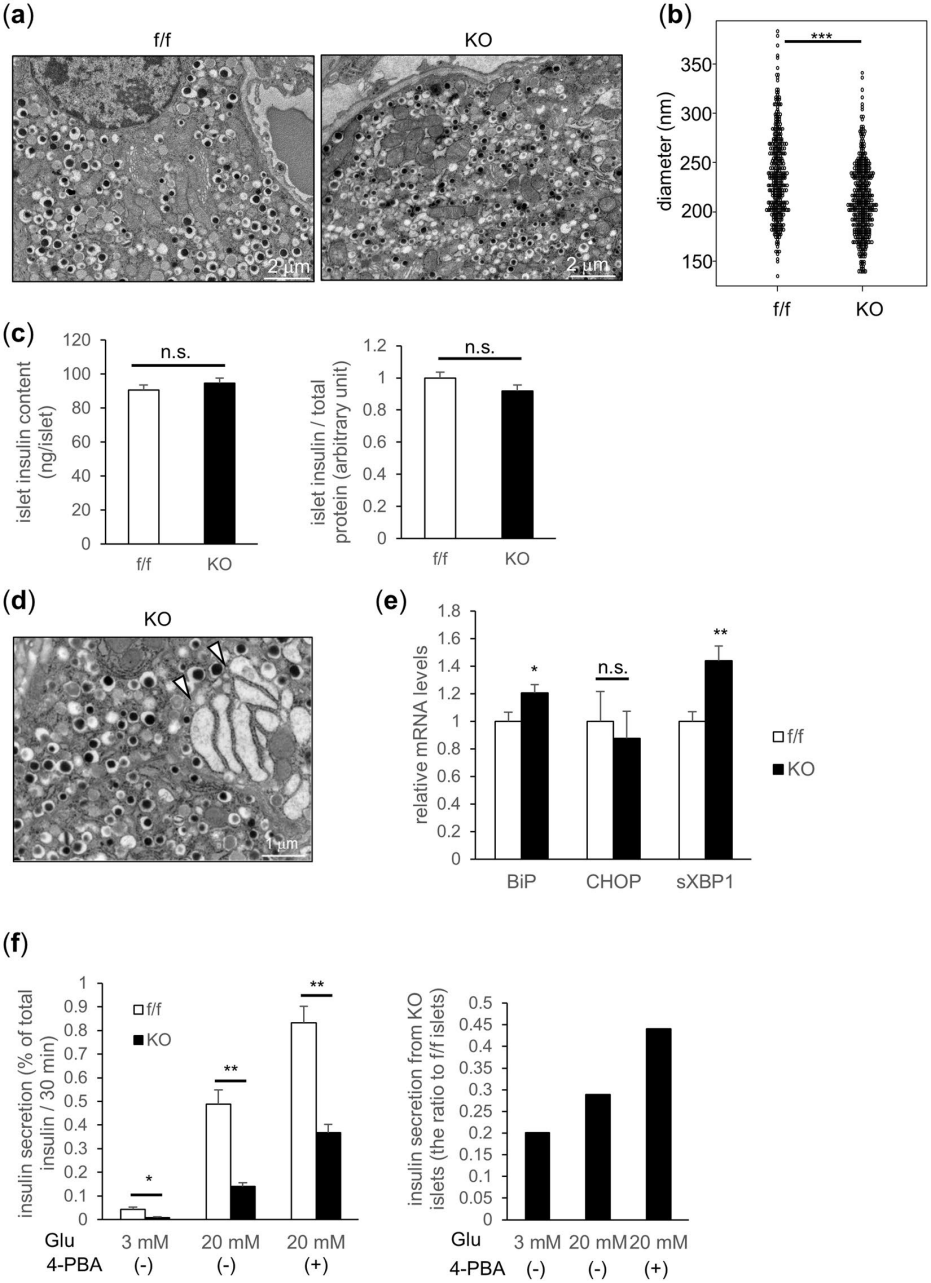
TFG depleted β-cells display smaller insulin crystals and mild ER stress. (**a**) Representative electron microscopic images of β-cells from 10-week-old f/f and KO mice (scale bars: 2 μm). (**b**) Insulin crystal diameter inside the granules (f/f: n = 377, KO: n = 435). (**c**) Islet insulin content (19-week-old mice, n = 8) (left: ng/islet, right: adjusted by the protein concentration). (**d**) Dilatation of the ER lumen observed in TFG KO β-cell (scale bar: 1 μm). White arrowheads indicate dilated rough ERs. (**e**) mRNA levels of ER-stress markers in isolated islets (10-week-old mice, n = 6). (**f**) Glucose-induced insulin secretion from isolated islets treated with or without a chemical chaperone (4-PBA) (20-week-old mice, n = 3–5). The ratio of insulin secretion from KO islets to that from f/f islets is shown in the right panel. *P < 0.05, **P < 0.01, ***P < 0.001.

Electron microscopic images of β-cells are shown in Fig. 6a. Crystal diameters of insulin granules were slightly but significantly smaller in β-cells of βTFG KO than in those of control mice (f/f 238 nm vs KO 212 nm (P < 0.001)) (Fig. 6b), which was presumed to partially account for the impaired insulin secretion observed in TFG depleted β-cells. No significant differences in islet insulin contents were detected (Fig. 6c).

Of note, ER swelling was often observed in β-cells of βTFG KO mice (Fig. 6d, Supplementary Fig. S2a,b), suggesting the existence of ER overload in these cells. Although no apparent upregulation was detected in the protein levels of the unfolded protein response (UPR) markers (Supplementary Fig. S2c), the mRNA levels of UPR markers, such as BiP and spliced XBP-1, were upregulated in TFG-depleted islets (Fig. 6e), which also indicates the presence of mild ER stress. To quantify the contribution of ER stress to the impaired insulin secretion capacity in TFG-depleted β-cells, isolated islets were incubated with a chemical chaperone, 4-phenylbutyrate (4-PBA), which alleviates ER stress, and subjected to the glucose-induced insulin secretion assay. 4-PBA restored, though not fully, the glucose-induced insulin secretion capacity of TFG-depleted islets from 28.9 to 44.1% of that in control islets (Fig. 6f), suggesting that the mild ER stress observed in TFG-depleted islets partially explains the impaired β-cell function in βTFG KO mice.

### Microarray analysis revealed attenuated NF-E2 related factor 2 (Nrf2) activation in TFG depleted islets

**Figure 7:**
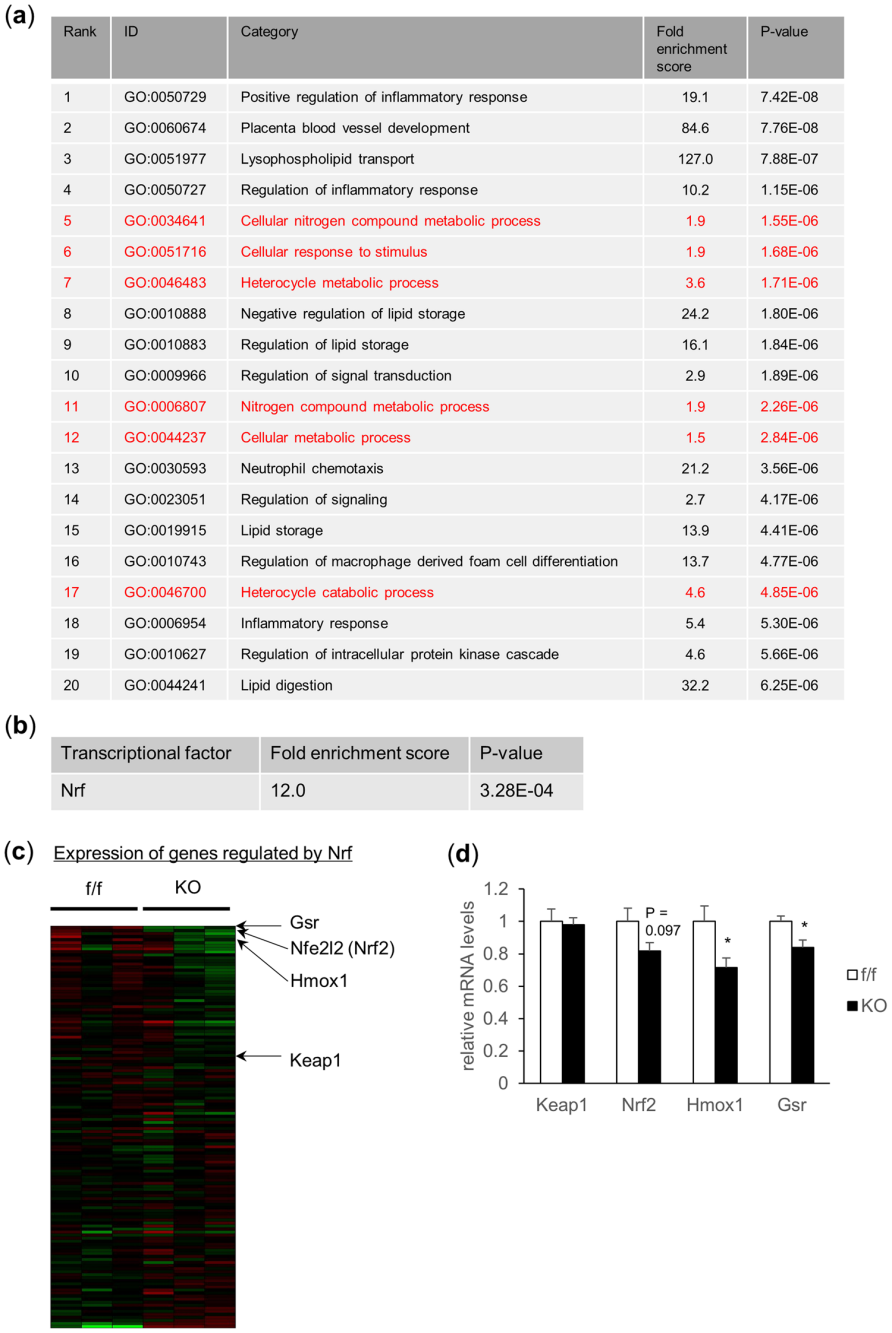
Microarray analysis revealed downregulation of the Nrf2 pathway in islets of TFG KO mice. Total RNA extracted from isolated islets of 10-week-old f/f and KO mice were subjected to microarray analysis. (**a**) Gene Ontology (GO) analysis. Top 20 GO terms significantly associated with 97 genes, extracted as described above, are shown. GO terms to which Nrf related genes (56 genes) are annotated with statistical significance (P < 0.05) are shown in red. (**b**) Transcriptional factors associated with expressions of genes which showed altered expression levels, differing by more than 1.25-fold between f/f and KO islets with statistical significance (P < 0.05), were analyzed using Keymolnet Lite software. Nrf was the only transcriptional factor identified in the analysis. (**c**) Heat map presentation of genes regulated by Nrf. (**d**) mRNA levels of genes in the Keap1-Nrf2 pathway in isolated islets (10-week-old mice, n = 6).

To further elucidate the mechanisms underlying the β-cell mass and/or function impairments in βTFG KO mice, total RNA extracted from isolated islets was subjected to microarray analysis to detect differences in gene expression patterns between islets of βTFG KO and control mice. Gene Ontology (GO) analysis was performed targeting 97 genes which showed different expression levels between f/f and KO islets (Fig. 7a).

Analysis using KeyMolnet Lite software revealed NF-E2 related factors (Nrf) to be possible transcriptional factors regulated downstream from TFG (Fig. 7b,c). Nrf was also among the transcriptional factors associated with TFG expression in our previous microarray analysis using LNCaP cells (a prostate cancer cell line) (Supplementary Table S1). The top 20 GO terms are shown in Fig. 7a, and several are also associated with Nrf related genes.

Among the Nrf genes, Nrf2 is a transcriptional factor which is activated in response to oxidative stress and induces the transcription of genes encoding antioxidant proteins^14^. β-cell protective functions of Nrf2 have been suggested in several reports describing pancreatic islets of *Nrf2*−/− mice being significantly reduced in size and showing impaired insulin secretion^15^, as well as Nrf2 activation by β-cell specific knockout of Kelch-like ECH-associated protein (Keap1), which interacts with Nrf2 and suppresses its activity by ubiquitination and subsequent Nrf2 degradation, providing protection against reductions in β-cell mass and insulin secretion capacity via oxidative stress^16^. Consistent with the microarray analysis findings, quantitative real-time PCR revealed downregulation of *Nrf2* and its target genes (such as heme oxygenase 1 (*Hmox1*), glutathione-disulfide reductase (*Gsr*)) in islets of βTFG KO mice (Fig. 7d, Supplementary Fig. S3a), which might partially account for the β-cell mass and function impairments in βTFG KO mice.

## Discussion

**Figure 8:**
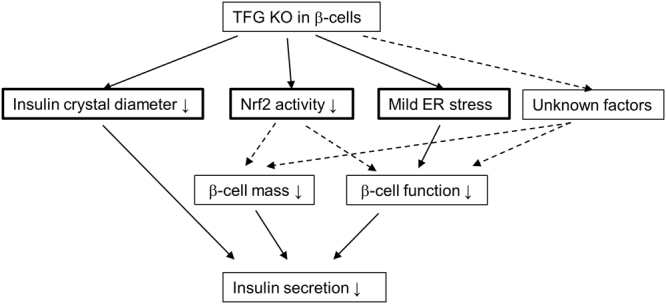
Effects of TFG depletion in pancreatic β-cells. TFG depletion in pancreatic β-cells resulted in smaller insulin crystal diameters, lower Nrf2 activity and mild ER stress. Downregulation of Nrf2 activity might partly account for the lower β-cell proliferation and the resultant β-cell mass reduction. Although the smaller insulin crystals and mild ER stress partially account for the impaired insulin secretion from TFG-depleted islets, additional factors, such as impairment of insulin granule recruitment, probably link TFG depletion and impaired β-cell function.

In this study, we demonstrated that TFG-deficiency leads to reduced β-cell mass, impaired insulin granule formation and suppressed glucose-stimulated insulin secretion from isolated islets. Thus, our results clearly show TFG in β-cell to control insulin secretion *in vivo* by multiple mechanisms (Fig. 8), which could at least partially explain the high coincidence of diabetes mellitus and HMSN-P.

The β-cell mass reduction in βTFG KO mice is very likely due to diminished β-cell proliferation rather than increased β-cell apoptosis. This is in line with a previous report which indicated that siRNA-mediated TFG knockdown inhibited cell growth in prostate cancer cell lines via induction of cellular senescence^17^. In addition, TFG-1 was shown to be essential for normal cell-size control in *C. elegans*, suggesting the cell-growth-promoting functions of TFG^18^. Although the precise mechanisms underlying the impaired β-cell proliferation remain unknown, downregulation of Nrf2 activity observed in TFG-depleted β-cells might partly account for the reduced proliferation. A role of Nrf2 in cell proliferation was proposed in a recent review^19^ and another report suggested G2/M arrest via Wee1 upregulation to be a cause of delayed liver regeneration in Nrf2-deficient mice^20^. In addition, upregulation of genes involved in the pentose phosphate pathway by Nrf2 has been shown to be a favorable metabolic adaptation to the excessive proliferation of cancer cells^21^. In TFG-depleted islets, although the differences were not striking, being slightly or marginally significant, upregulation of Wee1 and downregulation of genes encoding enzymes in the pentose phosphate pathway (glucose-6-phosphate dehydrogenase X-linked, phosphogluconate dehydrogenase) were observed (Supplementary Fig. S3b), indicating proliferation inhibitory signals to be activated and to thereby suppress proliferation. Of note, the impairment of β-cell mass in βTFG KO mice was more evident in both younger mice (Fig. 3c) and adult mice fed the HFHS diet (Fig. 4c) than in adult mice fed the NC diet (Fig. 4c), raising the possibility that the impaired β-cell proliferation manifests only when there is an increased demand for proliferation, such as in the developmental stage or as obesity develops, though further studies using a sufficient number of mice are needed to clarify this issue.

It has been recognized that excessive ER stress in pancreatic β-cells causes β-cell loss through ER stress-mediated apoptosis. Since the importance of TFG in transport from the ER to the Golgi apparatus via COPII vesicles has been confirmed^3^, it is reasonable to speculate that TFG depletion aggravates ER stress and thereby leads to β-cell loss. Indeed, prior reports have suggested that TFG depletion might lead to ER stress development and the resultant apoptosis^4,22^. Dilated ER and moderately elevated mRNA levels of UPR markers (Bip and spliced XBP-1) were observed in our study, indicating the existence of ER stress in TFG-depleted β-cells. Insulin secretion capacity was partially restored in 4-PBA treated βTFG KO islets, which also suggests that the observed impairment of β-cell function in βTFG KO mice is partially attributable to the increase in ER stress. However, no apparent β-cell apoptosis increase was histologically observed in either NC- or HFHS-fed β-cell specific TFG knockout mice, which was further confirmed by cleaved caspase-3 also being undetectable in βTFG KO islets in the Western blot analysis (Supplementary Fig. S2c). Therefore, we consider ER stress to be rather unlikely as a major cause of the impaired β-cell mass characteristic of β-cell specific TFG knockout mice. Why the ER stress demonstrated by dilated ER did not increase apoptosis is currently unclear. Although data obtained from isolated islets might underestimate the situation *in vivo*, since isolated islets are cultured in medium overnight before analysis, given that upregulation of the mRNA and the protein levels of UPR markers was not marked, the simplest explanation is that ER stress in TFG-depleted β-cells was mild and did not exceed the threshold for triggering apoptosis. It should be noted, however, that mRNA levels of C/EBP homologous protein (CHOP), which serves as a pro-apoptotic factor in ER stress induced apoptosis^23,24^, remained unchanged in TFG-deficient islets. This raises the alternative possibility that the PKR-like endoplasmic reticulum kinase (PERK) initiating axis of UPR was specifically inactivated by TFG deficiency, despite the activating transcription factor-6 (ATF6) and inositol requiring enzyme 1 (IRE1) axes being activated. Nrf2 activation is suggested to be downstream from PERK^25^. However, as discussed above, Nrf2 is not activated instead being downregulated in TFG-deficient islets, again showing no activation of the PERK axis of the UPR under TFG-deficiency.

Insulin secreted from β-cells has been recognized to stimulate β-cell proliferation in an autocrine manner^26^. Islet amyloid polypeptide (IAPP), which is co-secreted with insulin from β-cells, has also been demonstrated to promote β-cell proliferation through Erk1/2 and Akt activation at least under normoglycemic conditions^27^. Therefore, reduced autocrine actions of insulin and IAPP constitute another possible explanation for the reduced β-cell proliferation in βTFG KO mice. This mechanism seems unlikely, however, considering the lack of any apparent decline of Akt activation in βTFG KO islets (Supplementary Fig. S3c).

We observed that insulin crystal diameter was slightly but significantly smaller (by about 11%) in β-cells of KO mice than in those of control mice, although neither insulin mRNA (Supplementary Fig. S3d) nor islet insulin content was decreased in TFG-depleted islets. Since TFG is suggested to play an important role in vesicle transport, loss of TFG might have resulted in increases in immature vesicles with reduced insulin content per vesicle. Impairment of proinsulin ER export and its conversion to mature insulin was reported in MIN6 cells and isolated islets overexpressing the defective mutant Sar1, the activation of which initiates COPII coat assembly and is crucial in COPII vesicle formation^28^. Sar1 mutant overexpressing β-cells showed drastically distended ER and upregulation of UPR markers including CHOP. In contrast to Sar1, one of the three components (Sar1, Sec. 23/24, Sec. 13/31) required for *in vitro* reconstitution of COPII vesicle formation^29,30^, TFG is reportedly not required for the formation of COPII vesicles^3^ and the effects of TFG depletion on vesicle transport and secretion remain controversial. Audhya *et al*. showed that, upon TFG depletion, the tight association between ER and ERGIC was lost and the rate of cargo secretion from the ER was impaired^3,4^, whereas Stephens *et al*. demonstrated that TFG depletion resulted in small ERES and thereby disturbed the export of only large cargoes such as procollagen from the ER, but that the export of many other small cargoes together with the juxtaposition of ER and ERGIC remained intact even without TFG^5^. Further studies are necessary to elucidate the mechanisms underlying the smaller insulin crystals observed in TFG-depleted β-cells.

Glucose- or glibenclamide-induced insulin secretion from isolated islets was decreased by more than 60% in TFG-depleted islets, indicating defects in metabolism-secretion coupling in β-cells from TFG-deficient mice. It should be noted that neither ATP generation nor the intracellular calcium response to glucose was impaired in KO β-cells, suggesting defects in the very final signaling steps. Insulin granules that are reportedly already fused to the plasma membrane (termed ‘Old face’ granules) were released upon KCl stimulation, whereas insulin granules were newly recruited to and immediately released from the plasma membrane (termed ‘Restless newcomer’ granules) upon stimulation with glucose or sulfonylurea agents^31–33^. Since KCl-induced insulin secretion was less impaired than glucose- or glibenclamide-induced insulin secretion, it would be intriguing to examine whether the recruitment of insulin granules to the plasma membrane is disturbed by TFG depletion. However, further studies are needed to clarify this issue.

Clinically, a high incidence of glucose intolerance (7 with non-insulin-dependent diabetes mellitus (NIDDM) and 4 with impaired glucose tolerance (IGT) among 16 patients) was reported in patients with HMSN-P^13^, and two heterozygous missense mutations of TFG have been identified to date as being causative^6–8^. Given that the heterozygous TFG mutation is sufficient to cause HMSN-P and that TFG-positive cytoplasmic inclusions were observed in the motor neurons of one patient^6^, glucose intolerance in HMSN-P patients might be attributable to the cytotoxicity of abnormal TFG rather than to lack of TFG function as shown in our study. Whether patients with HSP, which is due to a homozygous mutation^10,11^ when TFG is the causative gene, have an associated glucose intolerance merits further study, but little is as yet known about this possible association.

Finally, it should be noted as an important limitation of our study that only isolated islets from βTFG KO were employed to establish the role of TFG in pancreatic β-cells. Data using other complementary approaches, such as siRNA-mediated TFG knockdown or TFG deletion using the CRISPR-Cas9 system in pancreatic β cell lines, are needed to validate our findings.

In conclusion, we have shown that TFG in pancreatic β-cells plays a vital role in maintaining both the mass and the function of β-cells. Since this is the first study, to our knowledge, using conditional TFG knockout mice, our results not only provide insights into the relationship between TFG and glucose metabolism, but also clues to understanding the pathogenesis of TFG-related neurodegenerative diseases.

## Methods

### Animals

Pancreatic β-cell specific TFG knockout mice were generated by crossing TFG floxed mice (TFG^loxP/loxP^, Accession No. CDB1026K: http://www2.clst.riken.jp/arg/mutant%20mice%20list.html) with β-cell specific Cre transgenic mice which express Cre recombinase driven by the mouse insulin promotor (MIP-Cre). TFG floxed mice were generated essentially as previously described^34^ (Fig. 1a). The genotyping primers used were P1 (ttg ttg att cag tga aag tgg gtg g) and P2 (ccc caa agt tgc ctc tcc ta) for both the wild and the floxed type allele. All mice used in this study were male, had a C57BL/6 background and were housed in temperature- and light-controlled rooms with free access to food and water. The high-fat-high-sucrose (HFHS) diet was from Research Diets (New Brunswick, NJ, USA) (D12327) and mice were fed the HFHS diet for 20 weeks starting at age 6 weeks. All animals were handled in accordance with the Guidelines for the Care and Use of Experimental Animals published by Hiroshima University and the RIKEN animal experimentation guideline, and all protocols were approved by the Institutional Review Board of Hiroshima University and the Institutional Animal Care and Use Committee of RIKEN Kobe Branch.

### Metabolic studies

For IPGTT, mice were fasted for 16 h and then received an intraperitoneal glucose injection (2 g/kg body wt). For the ITT, mice were fasted for 6 h and received an intraperitoneal insulin injection (0.75 units/kg body wt). Whole venous blood was obtained at the indicated times after the glucose or insulin load and then the blood glucose level was measured using Medisafe Mini (Terumo, Tokyo, Japan). Plasma insulin levels were determined using the Ultra Sensitive Mouse Insulin ELISA Kit (Morinaga Institute of Biological Science, Yokohama, Japan).

### Islet isolation and insulin secretion assay

Islets were isolated by perfusing the pancreatic duct with Collagenase P (Roche, Basel, Switzerland), followed by digestion for 12 min at 37 °C. Islets were picked manually and incubated in RPMI 1640 media overnight at 37 °C. For the insulin secretion assay, islets were incubated with HKRB buffer (135 mM NaCl, 3.6 mM KCl, 2 mM NaHCO_3_, 0.5 mM NaH_2_PO_4_, 0.5 mM MgCl_2_, 1.5 mM CaCl_2_, HEPES 10 mM, BSA 0.5% (w/v)) containing 3 mM glucose for 30 min and then HKRB buffer containing glucose, KCl or glibenclamide (Sigma-Aldrich, Darmstadt, Germany) was subsequently added. After centrifugation, supernatants were collected and insulin concentrations were measured using a Mouse Insulin ELISA Kit (Morinaga Institute of Biological Science, Yokohama, Japan). Pellets were also washed with phosphate buffered saline (PBS), solubilized with lysis buffer (50 mM Tris-HCl (pH 7.4), 150 mM NaCl, 1 mM EDTA, 1% Triton X-100) and subjected to insulin measurement. In the insulin secretion assay of 4-PBA treated islets, islets were incubated in RPMI media with 1 mM 4-PBA (Sigma-Aldrich) or with dimethyl sulfoxide (Wako, Osaka, Japan) overnight prior to glucose stimulation.

### Intracellular Ca^2+^ measurement

Isolated islets were placed on glass-based dishes coated with Cell-Tak (Corning, NY, USA) and then incubated overnight with RPMI media at 37 °C. Islets were incubated with 5 μM Fura-2 AM (Dojindo Laboratory, Kumamoto, Japan) for 30 min, followed by washing with HKRB buffer three times before measurement. AQUACOSMOS software (Hamamatsu Photonics, Hamamatsu, Japan) was used to detect fluorescence at 510 nm by excitation at 340 or 380 nm, and HKRB buffer containing either glucose or KCl was added 60 sec after the start of measurement.

### Islet ATP content measurement

Isolated islets (10 islets each) were incubated with HKRB buffer containing 3 mM glucose for 30 min and then stimulated with glucose for 15 min. Intracellular ATP content was measured using ATPlite (Perkin Elmer, Waltham, MA, USA). The data were normalized by the protein concentration measured using a Protein Assay BCA Kit (Wako).

### Islet insulin content measurement

Isolated islets (10 islets each) were washed once with PBS, solubilized with lysis buffer and subjected to insulin measurement. The data were normalized by the protein concentration of the lysate.

### Western blotting

Tissues were homogenized with lysis buffer (50 mM Tris-HCl (pH 7.4), 150 mM NaCl, 1 mM EDTA, 1% Triton X-100), centrifuged at 15,000 rpm for 10 min and subsequently for 30 min at 4 °C. After adjusting the protein concentrations, the supernatants were mixed and boiled with sample buffer. For islets, an equal amount of isolated islets was directly solubilized with sample buffer. Samples were electrophoresed with SDS-polyacrylamide gel, transferred to PVDF membranes and subjected to immunoblotting using Supersignal West Pico Substrate (Thermo Scientific, Waltham, MA, USA) or ImmunoStar LD (Wako). Antibodies were purchased from Abcam (Cambridge, MA, USA) (TFG (ab156866)) and Santa Cruz Biotechnology (Dallas, TX, USA) (tubulin (sc-5286)).

### Quantitative real-time PCR

Total RNA was extracted from isolated islets using NucleoSpin RNA XS (Macherey-Nagel, Düren, Germany) and first-strand cDNA was obtained using a Verso cDNA Synthesis Kit (Thermo Scientific), according to the manufacturer’s instructions. Real-time PCR was performed using the CFX96 real-time PCR system (Bio-Rad, Hercules, CA, USA) with a KAPA SYBR FAST qPCR Kit (KAPA Biosystems, Wilmington, MA, USA).

### Microarray analysis

Total RNA was extracted from isolated islets as described above. After checking the quality of the extracted RNA using Bioanalyzer 2100 (Agilent Technologies, Santa Clara, CA, USA), 150 ng of RNA were subjected to reverse transcription using Transcriptor Reverse Transcriptase (Ambion, Foster City, CA, USA) and hybridization onto Affymetrix Mouse Gene 1.0 ST microarray chipsets (Affymetrix, CA, USA). The arrays were scanned using the Affymetrix GeneChip Scanner 3000 7 G controlled by GeneChip Operating Software, 1.3. Genes which showed altered expression levels, differing by more than 1.25-fold between f/f and KO islets, with statistical significance (P < 0.05) were identified, and then the transcription factors associated with expressions of these genes were analyzed using KeyMolnet Lite (KM Data, Tokyo, Japan) as previously described^35^.

### Immunohistochemistry

Immunohistochemistry was performed as previously described^36^. Briefly, paraffin-embedded pancreatic sections were deparaffinized in xylene and rehydrated. Sections were incubated in 0.1% Triton for 5 min and then heated in 10 mM citrate buffer (pH 6.0) using a microwave for antigen activation. After blocking with 3% goat serum for 1 hr, sections were incubated with first antibodies [anti-TFG (Abcam), anti-insulin (Abcam), anti-glucagon (Cell Signaling Technology), anti-Ki-67 (Abcam)] overnight at 4 °C. Sections were then incubated with secondary antibodies conjugated with either fluorescein isothiocyanate (FITC) or Cy3 for 1 hr and then mounted with DAPI-containing mountants. Digital images were obtained with a multifocal microscope (BZ-9000; KEYENCE, Osaka, Japan). TUNEL stainings were performed using the DeadEnd Fluorometric TUNEL System (Promega, Madison, WI, USA) basically according to the manufacturer’s instructions, except that we double-stained with insulin to identify pancreatic β-cells. To quantify the β-cell mass, the images of all of the insulin positive areas in the section were obtained employing microscopy and the areas were quantified using ImageJ. Whole pancreas areas were determined using ImageJ by quantifying the area of the Hematoxylin-Eosin stained section and the β-cell area per pancreas was then determined as the percentage of the β-cell area to the whole pancreas area. To calculate the percentage of Ki-67 positive β-cells, approximately 10 images of islet(s) were randomly obtained from each mouse and the numbers of Ki-67 positive β-cell nuclei and total β-cell nuclei were counted manually.

### Electron microscopy

Pancreases from 10-week-old mice were fixed with 2.5% glutaraldehyde. The pancreases were cut into pieces and then fixed for 16 hr in 2.5% glutaraldehyde in phosphate buffer (pH 7.4) and subsequently treated with 1% osmium tetroxide for 1 hr at 4 °C. The specimens were dehydrated in a graded ethanol series and then embedded in Epon 812. Ultrathin sections were obtained using a diamond knife and then stained with uranyl acetate and lead citrate, and observed by transmission electron microscopy using JEM-1230 (JEOL, Tokyo, Japan) with a MegaView G2 CCD camera (Olympus, Tokyo, Japan) operated at 80 kV.

### Statistical analysis

Statistical analyses were performed using EZR (Saitama Medical Center, Jichi Medical University, Saitama, Japan). Values are presented as means ± SE. We used student’s unpaired t-test when comparing two groups, and one-way ANOVA followed by the post-hoc Tukey’s test for multiple comparisons, unless otherwise noted. We considered P < 0.05 to indicate a statistically significant difference.

## Electronic supplementary material

Supplementary Information
