# Supplementary material for: Trk-fused gene (TFG) regulates pancreatic β cell mass and insulin secretory activity
Source: Sci Rep. 2017 Oct 12;7:13026. doi: 10.1038/s41598-017-13432-x (PMC5638802; doi:10.1038/s41598-017-13432-x)
Supplement: Supplementary file 1 — Supplementary Information [file 41598_2017_13432_MOESM1_ESM.pdf]

Supplementary Information

Trk-fused gene (TFG) regulates pancreatic β cell mass and insulin secretory activity

Takeshi Yamamotoya, Yusuke Nakatsu, Akifumi Kushiyama, Yasuka Matsunaga, Koji Ueda, Yuki Inoue, Masa-Ki Inoue, Hideyuki Sakoda, Midori Fujishiro, Hiraku Ono, Hiroshi Kiyonari, Hisamitsu Ishihara, Tomoichiro Asano

Supplementary Figure S1

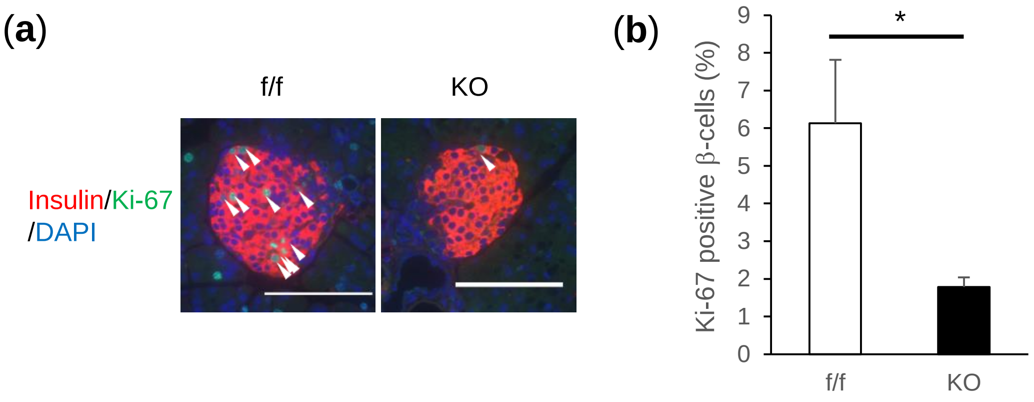

Supplementary Figure S1.

(**a**) Immunostaining for Ki-67 (green) and insulin (red). White arrowheads indicate Ki-67 positive β-cells. scale bars: 100 μm. (**b**) Percentage of Ki-67 positive β-cells (f/f: 406 positive cells among 8016 β-cells, KO: 227 positive cells among 10981 β-cells). 3-week-old mice, n = 6-10, *: $P < 0.05$.

Supplementary Figure S2

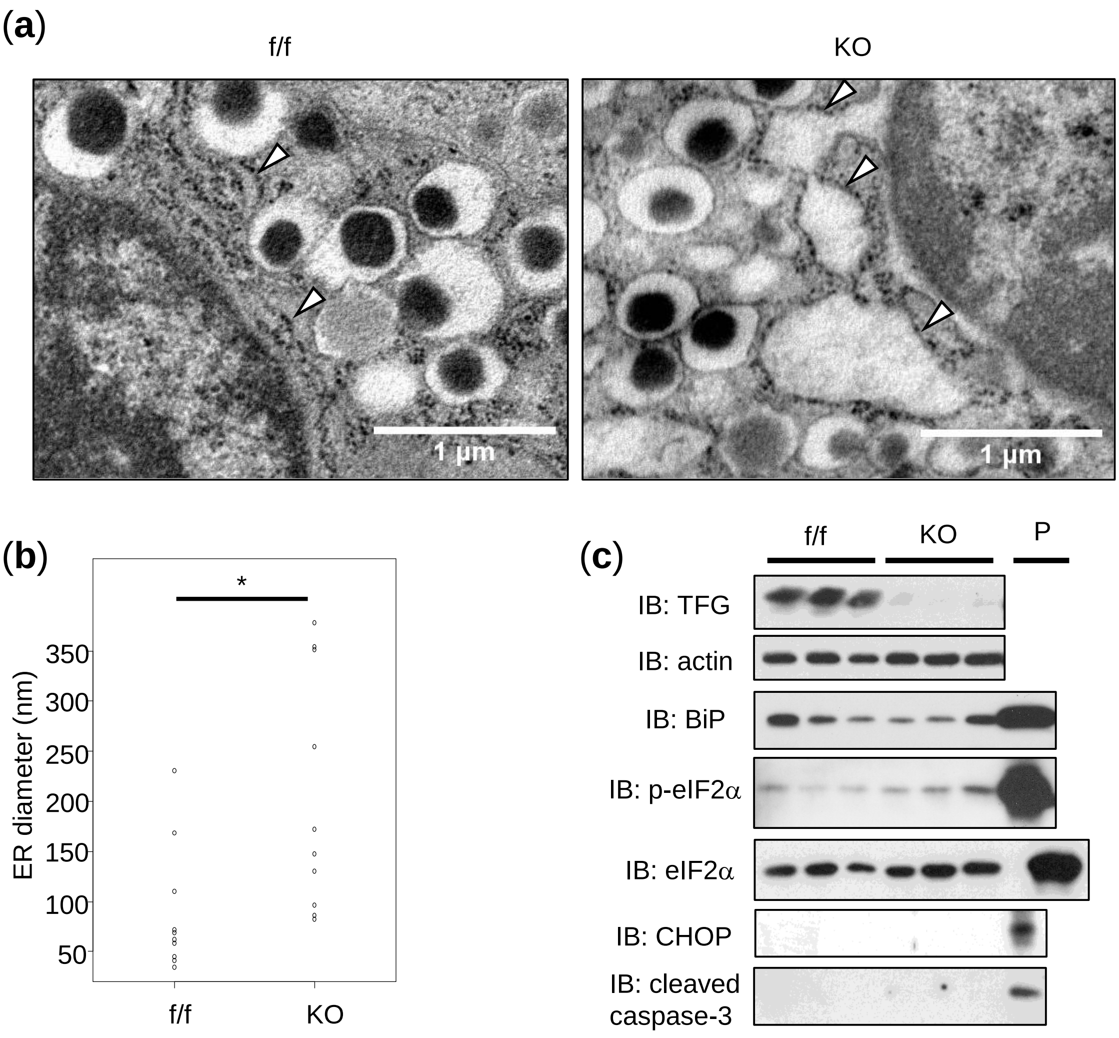

Supplementary Figure S2.

(**a**) Electron microscopic images of β-cells from f/f and KO mice (scale bars: 1 μm). White arrowheads indicate rough ERs, which showed dilatation in the β-cell from KO mice. (**b**) ER diameter of f/f and KO β-cells. Each prot represents the average of 5 measurements within an image. (**c**) Western blot analysis of ER stress markers and cleaved caspase-3 in isolated islets. P: positive control. *: $P < 0.05$.

For the Western blotting for ER stress markers and cleaved caspase-3, HEK-293 cells were treated with 1 mM Thapsigargin (Cayman Chemical, Ann Arbor, MI, USA) overnight and the cell lysates were used as a positive control. Antibodies were purchased from Cell Signaling Technology (Danvers, MA, USA) (BiP (#3177), CHOP (#2895), p-eIF2a (#3597), eIF2a (#5324), cleaved caspase-3 (#9661)) and Santa Cruz Biotechnology (actin (sc-47778)).

Supplementary Figure S3

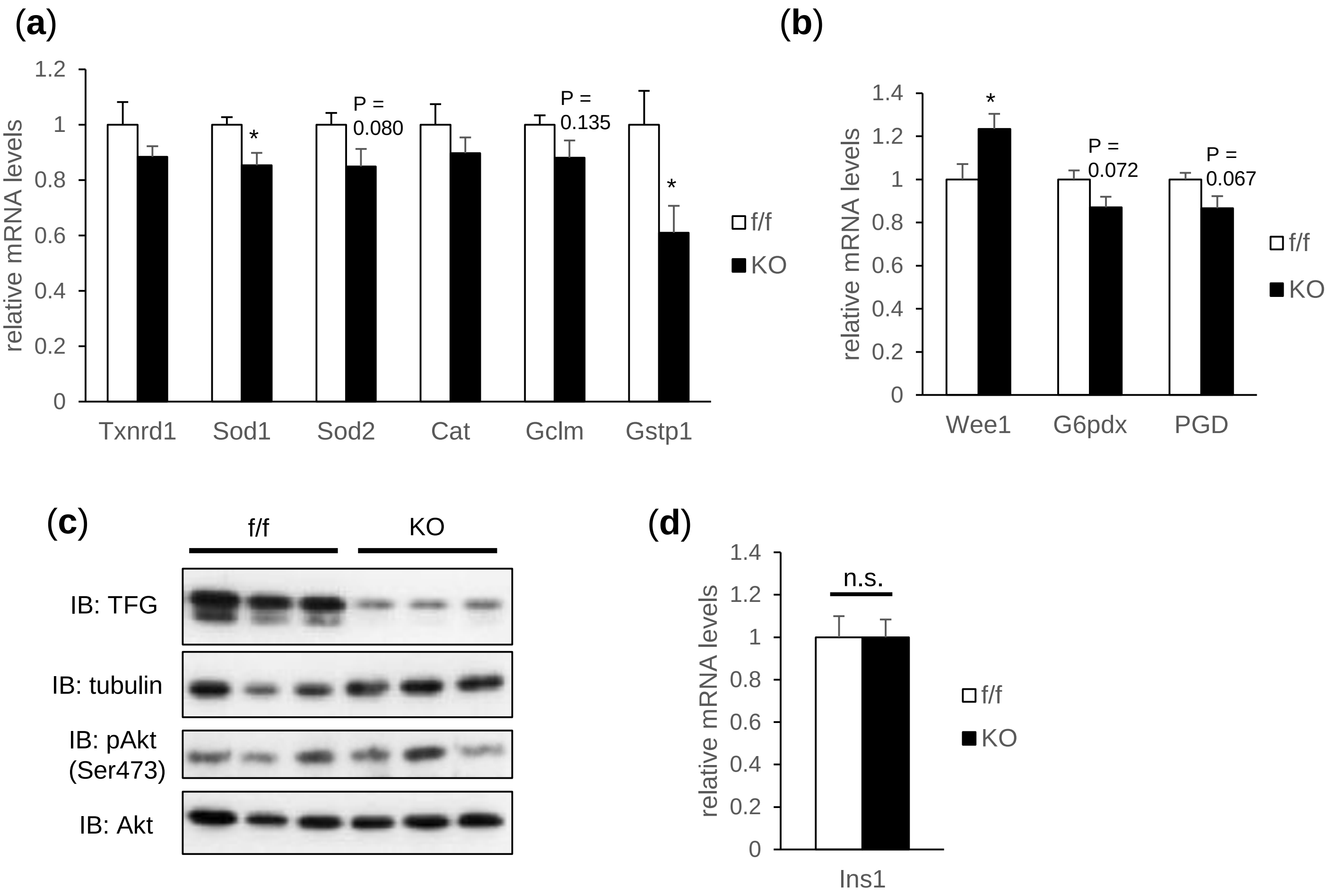

Supplementary Figure S3.

(**a**) mRNA levels of thioredoxin reductase 1 (Txnrd1), Superoxide dismutase 1 (Sod1), Superoxide dismutase 2 (Sod2), Catalase (Cat), Glutamate-cysteine ligase modifier subunit (Gclm) and Glutathion S-transferase P 1 (Gstp1) in isolated islets (n = 5-6). (**b**) mRNA levels of Wee1, glucose-6-phosphate dehydrogenase (G6pdx) and phosphogluconate dehydrogenase (PGD) in isolated islets (n = 6). (**c**) Western blot analysis of insulin signaling in isolated islets. (**d**) mRNA levels of Ins1 in isolated islets (n = 6). *: $P < 0.05$.

Antibodies were purchased from Cell Signaling Technology (pAkt (Ser473) (#4060), Akt (#4691)).

Supplementary Figure S4

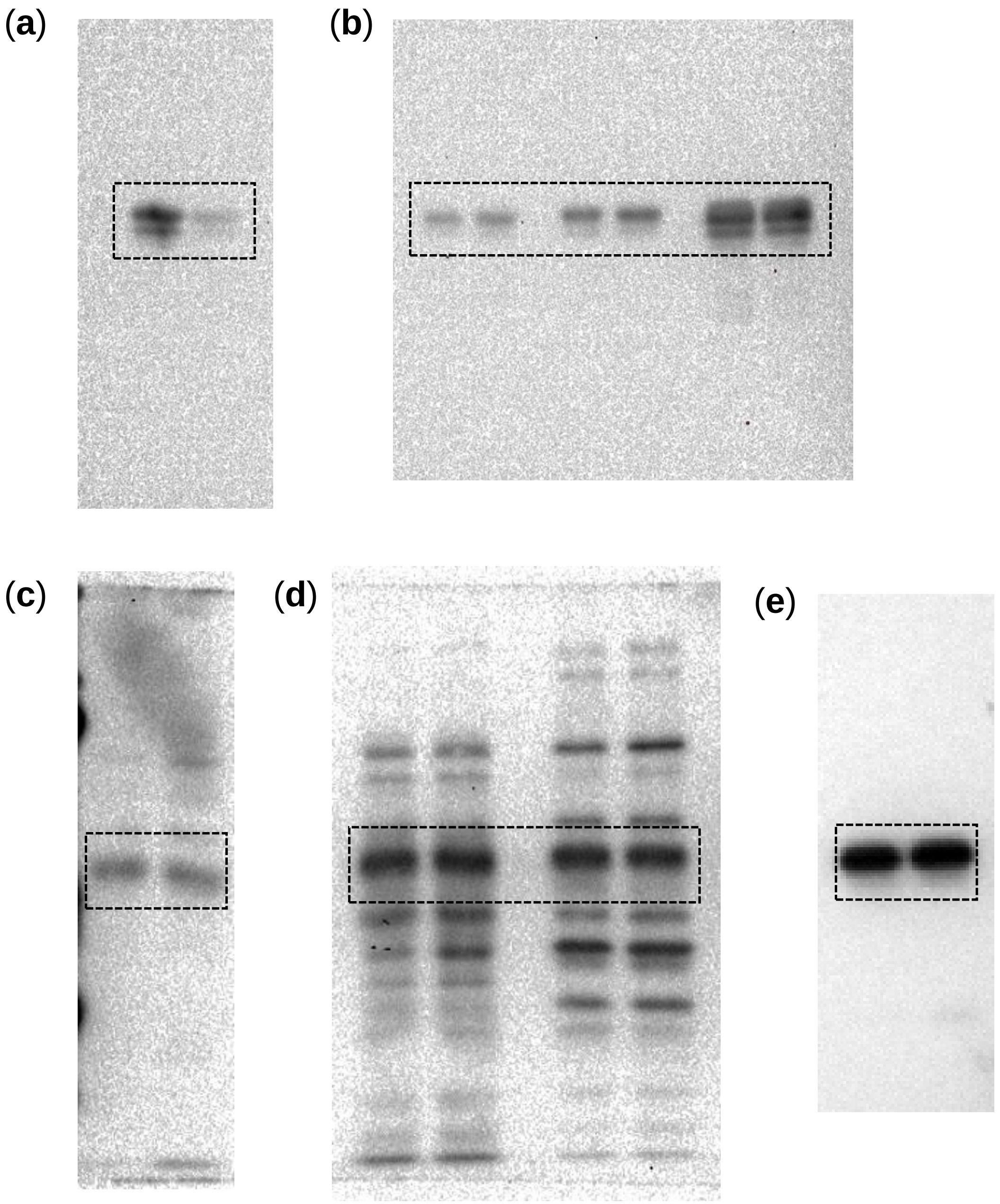

Supplementary Figure S4. Full-length blots for Figure 1b.

(**a**,**b**) Immunoblotting for TFG in samples from islet (**a**), liver, muscle and brain (**b**).

(**c**-**e**) Immunoblotting for tubulin in samples from islet (**c**), liver, muscle (**d**) and brain (**e**).

Supplementary Table S1

Transcriptional factor	P-value
MRF	2.43E-05
FOXO	6.00E-05
p73	5.01E-03
VDR	7.71E-03
MEF2	8.01E-03
p53	1.11E-02
Nrf	1.18E-02
Ternary complex factor	1.78E-02
C/EBP	1.79E-02
AP-1	3.98E-02
MTF-1	4.17E-02

Supplementary Table S1.

LNCaP cells (a prostate cancer cell line) were treated either with TFG siRNA or with control siRNA and subjected to microarray analysis as previously described[37]. Transcriptional factors associated with expressions of genes which showed altered expression levels, differing by more than 2-fold between cells treated with TFG siRNA and control siRNA are shown in the table.

Reference

37. Kanaoka, R. et al. Pin1 Inhibitor Juglone Exerts Anti-Oncogenic Effects on LNCaP and DU145 Cells despite the Patterns of Gene Regulation by Pin1 Differing between These Cell Lines. PLoS One 10, e0127467, doi:10.1371/journal.pone.0127467 (2015).
